# Exosomes and Other Extracellular Vesicles: The New Communicators in Parasite Infections

**DOI:** 10.1016/j.pt.2015.06.009

**Published:** 2015-10

**Authors:** Gillian Coakley, Rick M. Maizels, Amy H. Buck

**Affiliations:** 1 Institute of Immunology and Infection Research, School of Biological Sciences, University of Edinburgh, Edinburgh EH9 3FL, UK; 2 Centre for Immunity, Infection, and Evolution, School of Biological Sciences, University of Edinburgh, Edinburgh EH9 3FL, UK

## Abstract

Extracellular vesicles (EVs) have emerged as a ubiquitous mechanism for transferring information between cells and organisms across all three kingdoms of life. In addition to their roles in normal physiology, vesicles also transport molecules from pathogens to hosts and can spread antigens as well as infectious agents. Although initially described in the host–pathogen context for their functions in immune surveillance, vesicles enable multiple modes of communication by, and between, parasites. Here we review the literature demonstrating that EVs are secreted by intracellular and extracellular eukaryotic parasites, as well as their hosts, and detail the functional properties of these vesicles in maturation, pathogenicity and survival. We further describe the prospects for targeting or exploiting these complexes in therapeutic and vaccine strategies.

## Trends

EVs are secreted by most or all organisms and are emerging as a ubiquitous and functionally diverse type of host–pathogen interaction.

EVs contain a diverse suite of molecules including proteins, lipids, and nucleic acids, some of which are known to have immunomodulatory properties.

Parasite-derived EVs can communicate information and transfer genetic material to host cells or other parasites.

Host-derived EVs can play a key role in host defense and are candidates for generating a vaccine against pathogenic infection.

## Host–Parasite Interactions: Do We Know it All?

More than 1 billion people worldwide are burdened by parasitic disease, including malaria [1] and neglected tropical diseases such as leishmaniasis, Chagas disease and helminthiases (http://whqlibdoc.who.int/hq/2012/WHO_HTM_NTD_2012.1_eng.pdf), with most prevailing in developing regions such as eastern Asia, sub-Saharan Africa, and the Americas [2]. The prospects for drug resistance are alarming, with an increasing incidence in livestock that highlights a potential threat to the human population through zoonotic transmission as well as having strong economic and social implications [3]. There is a clear need for more efficacious therapies, which require an improved understanding of how these parasites adapt to, and manipulate, their host environment. Most parasites at some stage in their life cycle rely on the ability to communicate with one another and with their hosts, but the mechanisms underpinning this communication are still coming to light. Research in this area has largely focused on the soluble proteins secreted by parasites, many of which down-modulate the host immune response (reviewed in 4, 5). For example, in the case of helminths, the egg stage of *Schistosoma mansoni* secretes an omega-1 glycoprotein, demonstrated in several studies to promote type 2 helper (Th2) skewing of dendritic cells (DCs) and T cells during infection 6, 7. The immunomodulatory lipoprotein antigen B is secreted by *Echinococcus granulosus* and facilitates Th2 polarization and limits migration of neutrophils to the site of infection [8]. The ES-62 protein from *Acanthocheilonema viteae* has potent anti-inflammatory properties on mast cells [9]. Protozoan parasites similarly secrete a range of immunomodulatory molecules; for example, *Trypanosoma cruzi* mucins have been associated with suppression of active T cell immune responses by inducing arrest in the cell cycle [10]. Secreted parasite proteins have also been proposed to be involved in metabolic adaptation to the host environment [11] and tissue invasion, where proteases play a major role [12]. In the past 5 years, EVs have been revealed as another component of parasite secretion products that provide a previously unrecognized mechanism to package and protect a set of parasite cargo for uptake and integration into other cells. EVs are known to play a role in communication and genetic exchange between microbes [13]. The functional niches in which EVs operate in eukaryotic parasites and other pathogens are still emerging and are summarized in Table 1.

## Exosomes and Other Extracellular Vesicles: Origins and Functions

**Figure 1:**
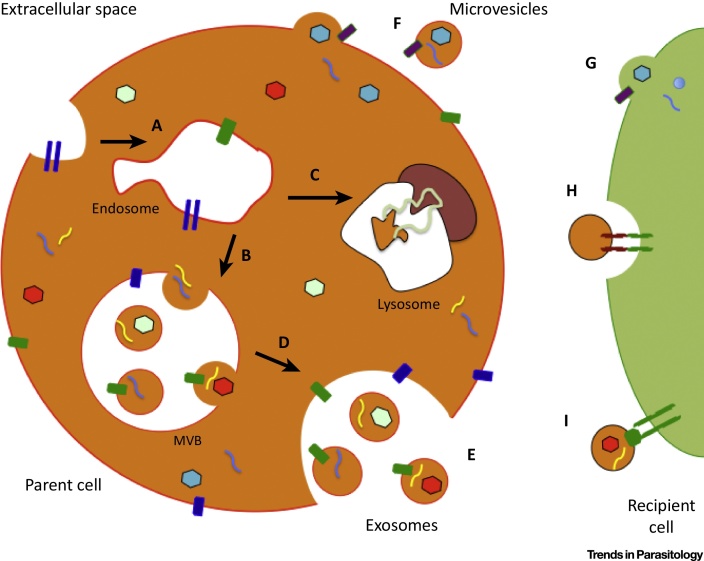
The Biogenesis and Transfer of Different Extracellular Vesicles (EVs). (A) Early endosome formation within the parent cell, surrounded by a range of different bioactive molecules [e.g., nucleic acids, proteins, lipids (denoted by different colors and/or shapes)]. (B) On development into a late endosome, inward budding allows capture of some of the host cell cytosolic contents in intraluminal vesicles (ILVs). The late endosome is also referred to as a multivesicular body (MVB). (C) Some mature MVBs fuse with the hydrolytic lysosome, where the vesicle cargo is subsequently degraded. (D) MVBs can also fuse directly with the plasma membrane, releasing their ILVs, now known as exosomes, into the extracellular space. (E) Release of exosomes into the extracellular environment. (F) Other microvesicles are released into the extracellular space following direct budding from the host cell plasma membrane. There are at least three mechanisms by which EVs interact with recipient cells: (G) direct fusion with the plasma membrane of the recipient cell; (H) receptor-mediated endocytosis following receptor–ligand interactions between EVs and the recipient cell; and (I) signaling via direct interactions of receptor and ligand on the recipient cell surface.

In mammalian systems EVs represent a mechanism of cell-to-cell communication through the direct stimulation of cells by receptor-mediated contact and/or through the transfer of genetic material, proteins, and lipids. Several distinct types of EV have been described, including those derived from the endocytic pathway, exosomes, versus those derived from shedding of the plasma membrane. We refer to the latter as microvesicles but note that these have been called by many names in the literature, including ectosomes, plasma membrane-derived vesicles, and microparticles [14]. Exosomes are endocytic vesicles approximately 40–100 nm in size that are released from most cell types [15]. Their biogenesis is initiated by inward budding of multivesicular endosomes (Figure 1). Consequently, exosomes express markers of their parent cells, but are also specifically enriched in other molecules associated with their biogenesis or that are selectively packaged into them; for example, by the endosomal sorting complexes required for transport (ESCRT) pathway (reviewed in [16]). Microvesicles can be difficult to distinguish from exosomes, but are generally up to 1 μm and bud from the plasma membrane, incorporating certain lipids, surface proteins, and other molecules before fission [17]. As reports in the literature do not always identify vesicular origin, here we refer to parasite exosomes or ‘exosome-like vesicles’ if they have been described as such in the primary literature or parasite microvesicles if they are suggested to derive from the plasma membrane, or ‘EVs’ if the origin is unclear.

In recent years, the literature surrounding EV function has exploded as their ubiquity in many biological and disease contexts has been realized [18]. Historically, these were first identified in reticulocytes as a mechanism to release transferrin receptors during maturation 19, 20 and then became of interest to immunologists as they contain MHCs and can present antigens [21]. However, following the report that functional mRNAs and miRNAs are transferred between mast cells via exosomes [22], there was further momentum in studying EVs as a mechanism of cell–cell communication. In this context, they have been shown to have various functions in immune cell activation and suppression 23, 24 and are also proposed to play a role in disease development and tissue homeostasis, [25]. An ever-expanding literature has also demonstrated various roles of EVs in diseases including cancer, since tumors also secrete these vesicles with oncogenes [26], such as those seen in gastrointestinal stromal tumor cell lines [27]. Exosomes and other EVs are now part of larger clinical initiatives to test their properties in drug delivery, their use as diagnostic biomarkers, and their potential as therapeutics. While most of this work has focused on oncology 28, 29, these vesicles also have exciting implications across a range of infectious diseases [30]. Here we detail the recent literature describing their roles in eukaryotic parasite infection, focusing on the communicative relationship between parasites and hosts. Furthermore we highlight the importance of EVs in the future identification of novel therapeutic targets and the development of vaccine strategies.

## Intracellular Protozoan Parasites: Host Manipulation by EVs

**Figure 2:**
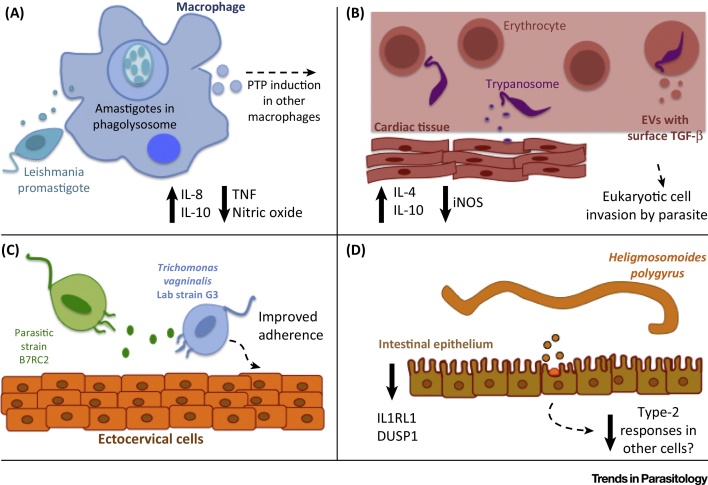
Schematic Representation of the Different Functions of Parasitic Extracellular Vesicles (EVs). (A) *Leishmania* spp. promastigotes release exosomes, which can modulate immune properties of monocytes, shown by an increase in the production of IL-8 and IL-10 and a decrease in tumor necrosis factor (TNF) and nitric oxide 35, 36, 37. Infected monocytes also release exosomes that have immunomodulatory properties in recipient cells (indicated by broken line), such as the induction of protein tyrosine phosphatases (PTPs) and changes in gene expression 39, 41. (B) *Trypanosoma cruzi* trypomastigote-shed microvesicles can induce type 2 helper (Th2) polarization [seen by an increase in IL-4 and IL-10 and a decrease in inducible nitric oxide synthase (iNOS)] and invasion of cardiac tissue (indicated by broken line) [34]. Infected erythrocytes and lymphocytes release microvesicles containing surface transforming growth factor beta (TGF-β) [47]. (C) The extracellular protozoan *Trichomonas vaginalis* secretes exosomes, which can promote better adherence of weaker strains to ectocervical cells [54]. (D) Adult *Heligmosomoides polygyrus* worms secrete exosomes as part of their excretory–secretory product in the lumen of the small intestine. These are potentially taken up by intestinal epithelial cells, where they modulate gene expression of the mitogen-activated protein (MAP) kinase regulatory phosphatase gene *dusp1* and the IL-33 receptor (ILRL1) and can have downstream suppressive effects on antiparasite type 2 responses [59].

Several protozoan parasites have been shown to release exosomes and/or microvesicles, including *Leishmania* species [31] and *T. cruzi* 32, 33, 34, the parasites that cause human leishmaniasis and Chagas disease, respectively. Seminal reports showed that promastigote and amastigote forms of *Leishmania donovani* and *Leishmania majo*r can release exosomes that are detected in host cells and selectively induce IL-8 secretion from macrophages 35, 36 (Figure 2A). The subsequent chemokinetic recruitment of neutrophils has been proposed as a ‘Trojan horse’ effect, whereby *Leishmania* can invade these cells and gain access to macrophages on phagocytosis of the infected neutrophils 37, 38. *Leishmania* exosomes have also been shown to induce the release of the immunosuppressive cytokine IL-10 and inhibit the inflammatory cytokine tumor necrosis factor (TNF) in human monocyte-derived DCs in response to interferon gamma (IFNγ). Pretreatment of mice with exosomes derived from *L. major* and *L. donovani* resulted in exacerbated infection and pathogenesis *in vivo*, associated with enhanced IL-10 production and a skewed Th2 response, preventing parasite expulsion as a type 1 response is normally required for clearance [37]. Specific components of *Leishmania* exosome cargo have also been identified and shown to be involved in immunomodulation, including elongation factor 1 alpha (EF-1α) and the membrane-bound metalloprotease GP63 [39]. These have both been associated with a depression in signalling events during a proinflammatory IFNγ response by monocytes (and potentially subsequent Th1 polarization) 36, 40. GP63 is also associated with numerous downstream modulatory effects during *Leishmania* infection, including the modulation of inflammation by activating macrophage protein tyrosine phosphatase (PTP) signaling. This metalloprotease has also been shown to impact protein sorting into exosomes and to inhibit miRNA processing in host cells by targeting the endoribonuclease DICER 39, 41, 42.

At least two types of EV have been identified from the infective (metacyclic trypomastigote) and noninfective (epimastigote) forms of *T. cruzi* parasites; both forms release microvesicles from the plasma membrane as well as exosomes presumed to derive from the endocytic pathway [32]. Following their initial identification [43], these EVs were further shown to contain a cohort of proteins associated with immune modulation and virulence and include the homolog to the multifunctional metalloprotease GP63, described above [32]. Notably, following inoculation of the parasite microvesicles and subsequent infection with *T. cruzi*, mice develop heightened cardiac parasitism and increased inflammatory infiltrates associated with higher levels of IL-4 and IL-10 [34]. These cytokines induce the polarization of a Th2 response as well as lower levels of inducible nitric oxide synthase (iNOS) in the tissue, suggesting that these microvesicles may serve to promote parasite dissemination and enhance survival (Figure 2B). Acid phosphatases involved in the adherence and infection of various trypanosome strains have also been shown to be present in the microvesicles [44].

In addition to the direct secretion of exosomes and microvesicles by these parasites, both *Leishmania* spp. and *T. cruzi* induce the release of exosomes from the cells that they infect. A study of *Leishmania mexicana*-treated macrophages *in vitro* showed that exosomes released from infected cells are capable of inducing phosphorylation of signaling proteins and significantly upregulating immune-related genes including adenosine receptor 2a (Adora2a) on macrophages [40]. Interestingly, Adora2a receptor activation on these cells by *Escherichia coli*, another pathogen that drives type 1 immune responses, has been associated with increased IL-10 and down-modulated TNF [45]. Conversely, a recent study suggests that exosomes from *Leishmania amazonensis*-infected macrophages can prime other naïve macrophages to initiate antiparasitic Th1 responses through the release of the inflammatory cytokines IL-12, IL-1β, and TNF [46]. *T. cruzi* also induces the release of microvesicles from infected host cells, including lymphocytes and monocytes *in vitro* and erythrocytes *in vivo*. These microvesicles express surface transforming growth factor beta (TGF-β), which has been shown to facilitate eukaryotic cell invasion by the parasite and leads to maturation and continuation of the life cycle [47]. The microvesicles also protect extracellular life cycle stages of *T. cruzi*, including epimastigotes from the vector and trypomastigotes from ruptured cells, from complement-mediated attack, thus facilitating parasite invasion of host cells [48]. More specifically, monocyte-derived microvesicles develop a complex with the complement C3 convertase C4b2a on the parasite surface, limiting the interaction with its substrate C3. The inhibition of this crucial step prevents complement-mediated lysis, opsonization, and the release of anaphylatoxins, subsequently leading to increased parasite survival [47]. In an analogous manner, erythrocytes infected with the malaria parasite *Plasmodium falciparum* produce microvesicles that enhance dose-dependent secretion of proinflammatory cytokines such as IL-1β, IL-6, and IL-12 from monocytes following phagocytosis [49]. In the context of infection, it has been hypothesized that these cytokines may aid endothelial cell activation and erythrocyte sequestration. As with many immunomodulatory mechanisms, however, it can be difficult to distinguish whether vesicle secretion by host cells during infection is controlled by the host and/or the parasite, as both may benefit. This is discussed further later in this review.

## Interspecies Communication between Intracellular Protozoan Parasites

In addition to manipulation of the host immune response, EVs can also mediate intercellular communication between parasites. It has been reported that microvesicles traffic between *P. falciparum*-infected erythrocytes and increase the commitment of asexual parasites to the sexual stages, gametocytes, to promote transmission 49, 50. Furthermore, it is suggested that EVs (described by Regev *et al*. as ‘exosome-like’ [50]) secreted by red blood cells following infection with transgenic *P. falciparum* parasites can rescue parasitic growth by transferring DNA encoding a drug resistance marker to other *P. falciparum* in infected cells under conditions of drug selection. Thus, genetic material can be transferred between the infected erythrocytes via EVs, and this may also contribute to the sexual development mentioned above. This pathway has been shown to be dependent on trafficking mechanisms that transport parasite-encoded proteins to the host-erythrocyte membrane through membranous structures called Maurer's clefts in infected erythrocytes [50].

This is one of the few examples to date of vesicle involvement in parasite-to-parasite communication (a further example is provided below in the case of the extracellular parasite *Trichomonas vaginalis*). This is likely to represent a bias in the literature, which focuses largely on the immunomodulatory properties of parasite secretion products. In the microbial context, it is well established that secreted vesicles play a role in microbe–microbe communication and genetic exchange (reviewed in [13]). Many gaps remain in our understanding of how different eukaryotic parasites communicate with one another to regulate aspects of their life cycles, including reproduction or commitment to transmission stages. It will be interesting to see whether this is a functional niche occupied by EVs that extends beyond malaria parasites.

## Extracellular Protozoan Parasites: Communication within Their Environment

An obvious function of EVs in extracellular pathogens is their ability to protect cargo and move this into host cells. However, mechanistic aspects of this are not understood, including whether and how there is specificity in the uptake by certain cell types, whether the parasite cargo is recognized by the host immune system, and how communication is conducted between two phylogenetically distant species. Among extracellular protozoan parasites, comparative analysis of the secretome of *Trypanosoma brucei* subspecies, the causative agent of African sleeping sickness, identified several exosome-associated proteins such as enolase, heat-shock protein 70, and the clathrin heavy chain. Different members of the metallopeptidase family are also found in the secreted microvesicles and may serve as potential drug targets or even diagnostic biomarkers during stages of African trypanosomiasis 51, 52. Complimentary studies on the *T. brucei* secretome also demonstrate the presence of 50–100-nm microvesicles budding from the plasma membrane of the infective parasite [53]. The parasitic protozoan *T. vaginalis*, which can cause infertility through sexual transmission, has been shown to release functional exosomes that can play a role in both parasite-to-parasite and parasite-to-host communication [54]. Virulence products are present within the exosomes that are able to specifically downregulate IL-8 secretion by ectocervical cells (potentially limiting neutrophil migration to prevent pathogen clearance). Furthermore, preincubation with exosomes released from a more adherent strain of the parasite, B7RC2, can induce better adherence of weaker strains, such the laboratory strain G3, in a dose-dependent fashion, which is not seen in the converse scenario (Figure 2C). The mechanisms underpinning these effects and the cargo within the exosomes involved are not yet known.

## Extracellular Parasites: Interactions at the Cell-to-Parasite Interface

**Figure 3:**
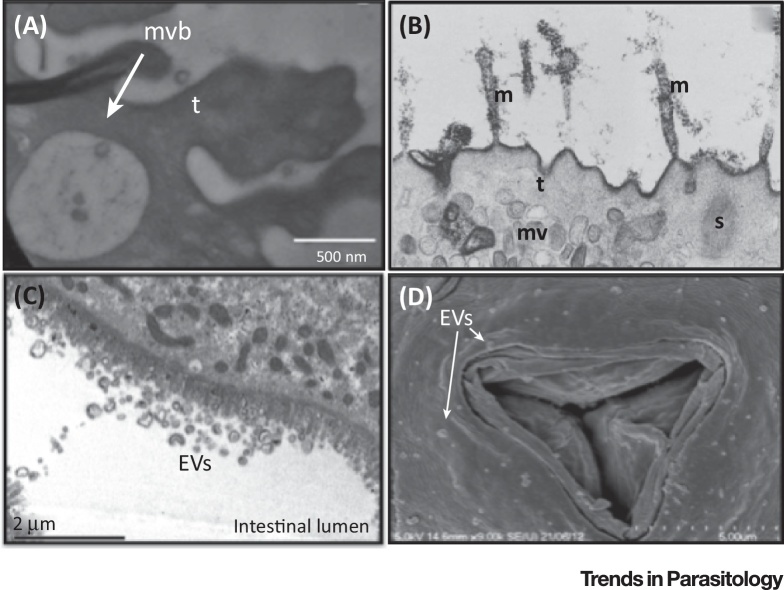
Electron Micrographs Demonstrating Exosome-Like Vesicles Derived from Extracellular Helminths. (A) Presence of exosome-like vesicles contained within the multivesicular body (MVB) on the tegument of *Echinostoma caproni*. Reproduced, with permission, from [56]. (B) Potential MVBs close to the tegumental surface of *Schistosoma mansoni* cercariae, microvilli (m), tegument (t), spines (s), and multilaminate vesicles (mv) are noted. Reproduced, with permission, from [57]. (C) Cross-section of *Heligmosomoides polygyrus* adult worms revealing numerous potential extracellular vesicles (EVs) within the intestinal lumen. Reproduced, with permission, from [59]. (D) Anterior opening of *H. polygyrus* covered in structures similar in size to exosomes, labeled as EVs.

Helminth worms are ubiquitous pathogens of plants and animals that have coevolved with their hosts for hundreds of millions of years and use sophisticated mechanisms for manipulating them [55]. It has only recently been demonstrated that these complex parasites also secrete exosomes, and potentially other classes of EV, into the environment that can be internalized by host cells. Electron microscopy images of EVs derived from diverse helminths are shown in Figure 3, including studies in the trematodes *Fasciola hepatica* and *Echinostoma caproni*, which release EVs that can be detected on the tegumental surface. Marcilla *et al.* [56], showed that these EVs are internalized by rat intestinal epithelial cells *in vitro* and contain protein homologs of proteins found in mammalian exosomes. Notably, earlier work examining the glycocalyx of *S. mansoni* cercariae demonstrated the potential presence of structures similar to multivesicular bodies adjacent to the schistosomula tegument [57]. A recent study has detailed the presence of exosome-like vesicles secreted by *Schistosoma japonicum* adults that were shown to induce macrophage polarization to a M1 phenotype, thereby highlighting the potential immunomodulatory properties of *Schistosoma*-derived exosomes and their potential role during infection [58].

We recently demonstrated that the gastrointestinal nematode *Heligmosomoides polygyrus* secretes exosomes that are internalized by host cells (Figure 2D). These are enriched in specific proteins, including those associated with exosome biogenesis (e.g., alix, enolase, HSP70), as well as many proteins of unknown function and contain miRNAs and other classes of noncoding RNA [59]. The presence of an Argonaute protein and small RNAs within nematode exosomes may suggest the existence of cross-species RNA interference, although the mechanism of this remains unknown. Several of the *H. polygyrus* exosome-derived miRNAs have target sites in the 3′ untranslated region (3′UTR) of the mouse *dusp1* gene, which encodes a mitogen-activated protein (MAP) kinase regulatory phosphatase. We showed that transfection of three nematode-derived miRNAs could suppress a luciferase reporter containing the 3′UTR of DUSP1. Although relatively little is known about this phosphatase in helminth infection, DUSP1^−/−^ macrophages have previously been shown to have sustained IL-10 expression in the presence of helminth cystatins [60]. IL-10 is an immunoregulatory cytokine that could prevent an antiparasitic or inflammatory response and promote parasite longevity within the host 61, 62. We further demonstrated that the *H. polygyrus* exosomes could suppress an inflammatory airway response *in vivo*: during the first 24 h of an innate atopic ‘danger’ response to the fungus *Alternaria alternata in vivo*, *H. polygyrus* exosomes block activation of type 2 innate lymphoid cells and have downstream effects on eosinophilic recruitment. Furthermore, *H. polygyrus* exosomes suppressed expression of IL1RL1/ST2 (the IL-33-specific receptor subunit) following treatment *in vitro* in intestinal epithelial cells and *in vivo* during the allergic asthma response to *Alternaria*. As the IL-33 ligand–receptor interaction is known to be important in antihelminthic responses 63, 64, these data suggest the ability of *H. polygyrus* exosomes to modulate aspects of the host cell response to prevent pathogen clearance. A previous report demonstrated that the model free-living nematode *Caenorhabditis elegans* releases peptide-containing exosomes using a defined apical secretion pathway [65] and it is expected that exosomes may be used by all nematodes, either as a mechanism of cell-to-cell communication within the organism or, when exported outside the organism, as a mode of communication with other species.

In addition to the above reports, analyses of the secretion products of other helminths suggest the presence of exosome-associated proteins, including CD63-like tetraspanins from the cestode *E. granulosus* [66]. Tetraspanins have been implicated in the formation and targeting of exosomes to recipient cells [67]. Interestingly, tetraspanins have independently been suggested as promising targets for vaccination against another parasite, *Echinococcus multilocularis*, the causative agent of alveolar echinococcosis 67, 68. This suggests that targeting exosomes and their surface proteins may provide an important antiparasite vaccination strategy.

## EVs from Microorganisms and Ectoparasites: More Players at the Extracellular Surface

Other eukaryotes, such as the pathogenic fungus *Paracoccidioides brasiliensis*, release highly immunogenic EVs that are detectable in the sera of paracoccidioidomycosis patients [69]. One such immunogenic epitope is the cellular membrane carbohydrate galactose-α-1,3-galactose (α-Gal), which is not found in human cells. Although α-Gal-enriched EVs may generate a robust immune response in the host, they are suggested to be beneficial to the pathogen, both by binding to host lectins and, potentially, by stimulating a suppressive type 2 response. This is in accordance with previous literature showing that α-Gal-enriched *T. cruzi* exosomes are able to stimulate IL-4/IL-10 expression in cardiac tissue and splenocytes [34]. Many types of opportunistic fungi, including *Cryptococcus neoformans*, *Candida albicans*, and *Histoplasma capsulatum*, release EVs [70], which have been suggested to contain virulence-associated factors including polysaccharides and lipids (reviewed further in [71]). The EVs released by *C. neoformans*, for example, are enriched in virulent capsular components such as glucosylceramide and glucuronoxylomannan (GXM) [72]. Interestingly, a recent study has shown the importance of phospholipid translocases (flippases) in *C. neoformans* exosome packaging and transport, whereby mutant Apt1 flippase-knockout fungi have diminished levels of GXM and are consequently unable to successfully colonize the lung and brain of infected mice [73]. Furthermore, the yeast *Malassezia sympodialis*, a component of natural human flora, is able to release EVs capable of generating IL-4 and TNF secretion from peripheral blood mononuclear cells, enhancing an inflammatory response in patients afflicted with atopic dermatitis [74]. Fungus-released EVs may also induce antimicrobial activity by host cells: EVs released by *C. neoformans* are taken up by macrophages *in vitro* and stimulate TNF, IL-10, TGF-β, and nitric oxide production [75].

A recent study in the argasid tick, *Ornithodoros moubata*, suggests that some immunomodulatory proteins may be secreted in arthropod saliva, and it is tempting to speculate that EVs would also be found in this environment. Proteomics of the tick saliva reveal several exosome-associated proteins (e.g., aldolase, enolase) as well as anti-inflammatory lipocalins, which serve as scavengers of leukotrienes, and adenosine nucleotides at the location of the bite [76]. It is clear that we are only at the beginning of many new discoveries with extracellular parasites and the functionally diverse EVs they might secrete. There are a growing number of reports containing proteomic matches to exosome proteins in parasite secretomes and this should cement the idea that these are probably used by most, or all, pathogens at some stage in their life cycle. The effects that these EVs may exert at this interface will be of particular importance in the context of antiparasite treatment, and conversely, based on the ability to suppress an innate immune response [59], they may also be useful tools to ameliorate inflammation-associated disease [4].

## Host Exosomes in the Context of Pathogen Infection: A Useful Therapeutic Strategy?

As parasites have evolved to secrete exosomes that are able to effectively interact with the host, it is only logical that the host would also use this pathway as a defense mechanism. During infection with a rodent malaria parasite, *Plasmodium berghei*, plasma cell-derived microvesicles induce CD40 on antigen-presenting cells, generating a potent inflammatory response through potential T cell priming and effector initiation [77]. Subsequently, macrophage activation may be responsible for clearance of the parasite. This is further supported by studies in *Plasmodium vivax* infection in humans, whereby immune cell-derived microvesicles are associated with greater acute inflammation in the pursuit of parasite eradication [78]. These mechanisms can be exploited in a therapeutic context; for example, murine reticulocytes infected with the nonlethal *Plasmodium yoelii* X strain can significantly attenuate pathogenesis when transferred into mice that are then infected with the lethal strain *P. yoelii* XL [79]. On a separate note, intestinal epithelial cells were shown to increase the release of antimicrobial peptide-containing exosomes in response to *Cryptosporidium* infection, which is driven by enhanced toll-like receptor 4 signaling following recognition of the protozoan parasite [80]. The facultative intracellular bacterium *Mycobacterium tuberculosis* induces exosome release from infected macrophages, which consequently promotes recruitment of lymphocytes through heightened inflammatory chemokine secretion (such as RANTES and MIP-1α) 13, 81. Exosomes derived from *Mycobacterium bovis*-infected macrophages are able to promote DC activation as well as generating an antibacterial T cell response *in vivo* [82].

Host-derived exosomes also play important roles in antigen presentation. DCs pulsed with *Toxoplasma gondii* antigens are able to induce both a systemic and a local humoral response against the parasite *in vivo*, thereby serving as an efficient vaccine against toxoplasmosis 83, 84. Similar results are seen in a vaccine trial with *L. major-*pulsed DC exosomes, showing that DC-derived exosomes are able to mediate protective Th1 immunity against cutaneous leishmaniasis in a cell-independent manner [85]. Importantly, several studies have emerged using DC-derived exosomes for protection against common livestock parasites. Vaccination of chickens with *Eimeria* parasite antigen-loaded DC exosomes was able to successfully ameliorate symptoms of avian coccidiosis caused by several species (*Eimeria tenella*, *Eimeria maxima*, and *Eimeria acervulina*) as well as reduce mortality rates [86].

## Concluding Remarks and Future Perspectives

From this review, it is clear that exosomes and other EVs can be used by both parasite and host to influence the outcome of an infection. Vesicles can function by transmitting signals between parasites, from parasite to host, or from host to the environment for antigen presentation and other aspects of host defense. The ability of vesicles to transport and deliver diverse populations of molecules in a specific package might occupy a range of niches in biology. There has been a surge of reports in the past 5 years detailing the presence of parasite-derived vesicles and it seems likely that this will only increase with the appreciation that all organisms are likely to secrete these [13]. Based on the literature, immune manipulation appears to be a prevalent function of parasite-derived exosomes, which feeds into numerous cell-to-cell interactions within the human body [87]. However, it is expected that EVs could also play a prominent role in parasite-to-parasite communication, which has been less well studied to date (see Outstanding Questions Box). The molecules within exosomes that mediate their functions require further investigation. We and others have detailed the small RNAs present in pathogen-derived exosomes 59, 88, 89, 90, 91 and previous reports have shown the functionality of exosomal RNA in an immune context 24, 92, 93. One concern in this field at present, however, is the lack of quantitative data to determine the abundance and stoichiometry of RNA within EVs and whether this is sufficient for effective gene silencing under physiological conditions [94]. Intriguingly, we found that an Argonaute protein is also secreted with exosomes derived from *H. polygyrus*, and it could be expected that ribonucleoprotein complexes, rather than individual molecules, might underpin functionality. In addition to nucleic acids, there are many immunomodulatory proteins in exosomes, 87, 95, 96, 97, as well as lipids that might have immunomodulatory properties [98]. During the preparation of this manuscript, two additional papers demonstrated EV secretion by helminths: the liver fluke *Opisthorchis viverrini* [99] and pig whipworm *Trichuris suis* [100]. Chaiyadet *et al*. [99] show that EVs produced by *O. viverrini* drive IL-6 production and proliferation of human cholangiocytes, and may link to the chronic periductal fibrosis associated with this pathogen. Additionally, they demonstrate that uptake of these EVs by host cells is blocked by Ab directed against a surface tetraspanin. A deeper understanding of the biochemical properties of exosomes will be key to interrogating how these complicated packages of information operate and how we can interfere with or mimic these processes to treat infectious disease.

Outstanding Questions How are the diverse combinations of molecules packaged into EVs integrated in a functional response in recipient cells?

Are all parasite EVs recognized by the host immune system or are they able to escape this?

How heterogeneous are the EVs secreted by parasites and is it possible that these have multiple targets and functions?

How is EV packaging and release regulated and can this be targeted as a therapeutic strategy?

What proteins are bound to the RNAs within EVs and how would these integrate into a functional RNAi pathway inside recipient cells?

## Figures and Tables

**Table 1:** Proposed Functions of Pathogen or Host-Derived Exosomes during Infection^a^

Pathogen	EV origin	Host or parasite?	EV target	Functional response	Effector mechanism	Refs
Protozoa						
*Leishmania amazonensis*	Macrophages exposed to promastigotes	Host	Monocytes and/or macrophages	Promotion of Th1 responses for parasite elimination	Naïve macrophages are primed to release IL-12, IL-1β, and TNF	[46]
*Leishmania donovani*	Promastigotes	Parasite	Monocytes and/or macrophages	Invasion and persistence within host cells and delivery of virulence factors	*Leishmania* EF-1α and GP63 activate host PTPs in monocytes responding to IFNγ. GP63 can also influence exosome cargo selection and inhibit host miRNA processing.	35, 36, 37, 39, 40, 42
		Parasite	Immune cells, including macrophages	Induction of *Leishmania* peptide-carrying exosomes from monocytes	Overall increase in IL-8 secretion by macrophages, which promotes neutrophil recruitment. Induces release of IL-10 in human monocytes while suppressing release of TNF.	35, 36, 37
*Leishmania major*	Promastigotes	Parasite	Monocytes/macrophages	Invasion and persistence within host cells and delivery of virulence factors	*Leishmania* EF-1α and GP63 activate host PTPs in monocytes responding to IFNγ.	39, 41
		Parasite	Immune cells, including macrophages and T cells	Increased disease exacerbation and Th2 polarization *in vivo*	Increase in the number of IL-4-producing CD4^+^ T cells/decrease in the number of IFNγ-producing CD4^+^ T cells	[37]
*Leishmania mexicana*	Macrophages exposed to promastigotes	Parasite	Macrophages	Immunomodulation of host signaling events promoting parasite survival	Upregulation of Adora2a by parasite-derived GP63 contained within host exosomes	[40]
*Plasmodium berghei*	Infected erythrocytes	Host	Macrophages	Activate systemic inflammation and T cell priming	Via MyD88/TLR4 pathway and CD40/CD40L interactions	[77]
*Plasmodium falciparum*	Infected erythrocytes	Parasite	Monocytes and macrophages	Transfer of parasite material and parasite dissemination	Innate cell activation. Cytokine induction in macrophages (IL-6, IL-12, IL-1β, and IL-10) in a dose-dependent manner.	[49]
		Parasite	Infected erythrocytes	Commitment of asexual parasites to gametocytes	Transfer of genetic information between parasites and budding of EVs via PfPTP2	49, 50
*Plasmodium vivax*	Platelets, erythrocytes, leukocytes	Host	Human immune cells, erythrocytes, endothelial cells	Higher acute fever and greater duration of malaria symptoms in human patients	Unknown mechanism	[78]
*Trichomonas vaginalis*	Mature parasites	Parasite	Ectocervical cells	Limit neutrophil migration to site of infection	Parasite-derived exosomes downregulate IL-8 secretion in ectocervical cells	[54]
		Parasite	Weakly adherent strains of the parasite	Promote adherence of weakly adherent strains and increase their virulence	Unknown mechanism	[54]
*Trypanosoma brucei*	Procyclic forms of the parasite (pathogenic in bloodstream)	Parasite	Host cells	Improved entry into host cells, enhanced parasite survival	Abundance of parasite-derived proteases (e.g., oligopeptidase B) favors parasite invasion	51, 52, 53
*Trypanosoma cruzi*	Trypomastigotes	Parasite	CD4^+^ T cells and macrophages	Th2 polarization leading to parasite dissemination and enhanced parasite survival	Increase in IL-4 and IL-10 secretion and reduction in iNOS expression in CD4^+^ T cells and macrophages	[34]
	Infected lymphocytes, monocytes and erythrocytes	Parasite	Recipient immune cells and monocyte-derived complement factors	Parasite invasion of host cells and inhibition of complement-induced parasite elimination	Plasma membrane-derived vesicles containing surface TGF-β, which promotes entry into host cells	47, 48
Fungi						
*Cryptococcus neoformans*	Exosomes secreted during the fungal cell phase	Pathogen	Host cells – unknown	Promote colonization of infected tissues	Release virulence factors – glucosylceramide and GXM	[72]
		Pathogen	Macrophages	Stimulate fungal killing	Enhanced IL-10 and TGF-β secretion and increased nitric oxide production by macrophages	[75]
*Malassezia sympodialis*	Yeast – skin-living flora component	Pathogen	PBMCs	Exacerbation of atopic dermatitis	Promote IL-4 and TNF secretion from PBMCs	[74]
*Paracoccidioides brasiliensis*	Yeast phase exosomes	Pathogen	Immune cells	Potential to skew to a suppressive Th2 response	Enriched in α-Gal, which may bind host lectins potentially improving infectivity by fungi	[69]
Helminths						
*Heligmosomoides polygyrus*	Intestinal tract of adult nematode	Parasite	Intestinal epithelial cells of the host	Suppress classical inflammation and danger responses, promoting parasite survival	Suppression of host targets including IL-33R and DUSP1	[59]
*Schistosoma japonicum*	Adult worms	Parasite	Macrophages	Polarization of host macrophages to M1 phenotype	Unknown mechanism	[58]

a Details in each column (from left to right) describe: the parasite species, the life stage and/or cellular origin of the EV, the proposed beneficiary (host or parasite), the proposed target and functional outcome, the mechanistic data in support of this function, and the primary literature reference.
